# Association of cardiovascular risk factors and intraplaque neovascularization in symptomatic carotid plaque

**DOI:** 10.3389/fneur.2024.1442656

**Published:** 2024-08-26

**Authors:** Zehao Liu, Lianlian Zhang, Bing Sun, Yasuo Ding

**Affiliations:** ^1^Department of Neurosurgery, The Affiliated Taizhou People’s Hospital of Nanjing Medical University, Taizhou School of Clinical Medicine, Nanjing Medical University, Taizhou, China; ^2^Department of Ultrasonography, The Yancheng Clinical College of Xuzhou Medical University, The First People’s Hospital of Yancheng, Yancheng, China

**Keywords:** plaque neovascularization, risk factor, atherosclerosis, vulnerable plaque, diabetes mellitus, cardiovascular risk factor

## Abstract

**Background and purpose:**

Cardiovascular risk factors are known to contribute to the formation of atherosclerotic plaques, which can result in carotid stenosis. However, the extent to which these factors are associated with intraplaque neovascularization, a key indicator of plaque vulnerability, remains unclear. To investigate this relationship, a study was conducted utilizing contrast-enhanced ultrasound (CEUS) to assess intraplaque neovascularization in symptomatic patients.

**Methods:**

A cohort of 157 symptomatic patients underwent evaluation using Contrast-Enhanced Ultrasound (CEUS) imaging to assess carotid intraplaque neovascularization, which was quantified based on the degree of plaque enhancement. The collected data encompassed baseline patient characteristics, results from biochemical examinations, cardiovascular risk factors, and medication usage history. Regression analyses were conducted to elucidate the relationship between carotid plaque neovascularization and various cardiovascular risk factors.

**Results:**

Patients with intraplaque neovascularization were more prone to have diabetes mellitus (OR 3.81, 95% CI 1.94–7.46, *p* < 0.001), dyslipidemia (OR 2.36, 95% CI 1.22–4.55, *p* = 0.011) and hypertension (OR 2.92, 95% CI 1.50–5.71, *p* = 0.002). Smoking increased the risk of having intraplaque neovascularization (OR 2.25, 95% CI 1.12–4.54, *p* = 0.023). Treatment with statins was significantly lower in patients with intraplaque neovascularization (OR 0.37, 95% CI 0.19–0.72, *p* = 0.003). In the multivariate analysis, diabetes mellitus (OR 3.27, 95% CI 1.10–9.78, *p* = 0.034) was independently related to the presence of intraplaque neovascularization. Meanwhile, compared to the patients in the first tertile of serum glucose (< 6.20 mmol/L), the patients in the third tertile (> 13.35 mmol/L) had the most significance of intraplaque neovascularization (OR 5.55, 95% CI 1.85–16.66, *p* = 0.002).

**Conclusion:**

The findings indicated that diabetes mellitus is a significant cardiovascular risk factor that is strongly associated with carotid intraplaque neovascularization.

## Introduction

Intraplaque neovascularization proliferation is a critical factor in the rapid progression of plaque and is closely associated with an increased risk of plaque vulnerability (1). When the tunica intima thickens beyond the oxygen diffusion limit, hypoxia within the plaque occurs. Chronic hypoxia can stimulate the sprouting of microvessels from the adventitial layer, which traverse the media and intima layers to reach the plaque (2). The vascular network within the plaque, characterized by a disorganized tree-like branching structure, actively contributes to metabolic activity by supplying nutrient-rich blood to the plaque. This network may serve as a significant indicator of plaque vulnerability through the cholesterol crystals deposition, necrotic core expansion, and inflammation infiltration (3). In contrast to normal vessels, the basement membrane structure of these abnormal physiological vessels is disrupted due to a deficiency in smooth muscle cells. Structural abnormalities and endothelial dysfunction result in newly formed microvessels that are fragile and leaky, prone to rupture (4). The rupture of hyperpermeable microvessels can result in intraplaque hemorrhage.

The association between atherosclerotic diseases and cardiovascular risk factors is well-established. High-risk cardiovascular factors are implicated in the development of atherosclerotic plaques, which may lead to carotid stenosis (5). Specifically, hypertension, diabetes, dyslipidemia, and elevated body mass index have been identified as contributors to carotid intima thickening, thereby facilitating plaque formation (6). Additionally, elevated systolic blood pressure, increased cholesterol levels, and smoking are closely associated with carotid artery stenosis. However, the relationship between these cardiovascular risk factors and the hallmark of plaque vulnerability—namely, intraplaque neovascularization—has been comparatively underexplored. Despite extensive research on intraplaque neovascularization in the context of plaque vulnerability, the specific risk factors associated with this process remain largely unidentified. Clinically, the identification of precise risk factors related to plaque neovascularization and the implementation of targeted interventions to mitigate plaque vulnerability could be pivotal in the prevention of stroke events. Identifying factors that contribute to plaque vulnerability may enhance risk assessment and facilitate personalized medical interventions.

Contrast-enhanced ultrasound (CEUS) represents a novel imaging technique for detecting intraplaque neovascularization (7). This method enables direct and dynamic visualization of microvessels within carotid plaque through the use of microbubble contrast agents (8). Studies have indicated that the semiquantitative evaluation of moving bright microbubbles *in vivo* can yield reliable information regarding the extent of plaque vessels (9). The observed augmentation in plaque enhancement was concomitant with a heightened risk of plaque vulnerability (10). The potential influence of cardiovascular risk factors on plaque neovascularization, which may accelerate the progression of carotid plaque vulnerability, remains to be elucidated. Consequently, this study seeks to investigate the relationship between plaque neovascularization and cardiovascular risk factors in symptomatic patients utilizing contrast-enhanced ultrasound.

### Study population

From January 2022 to February 2024, 330 consecutive symptomatic patients were enrolled in Stroke Centre. This retrospective study was approved by the Ethics Committee of Taizhou People’s Hospital (No. 2021-073-01), and all participants provided informed consent. Inclusion criteria: symptomatic patients with anterior circulation infarcts and at least one carotid plaque ipsilateral to the cerebral infarction. Symptomatic refers to stroke, transient ischemic attack, slurred speech, loss of control of a limb, or even hemiparesis. The thickest plaque ipsilateral to the cerebral infarction was selected as the culprit plaque. Exclusion criteria were patients unable to perform CEUS, plaques with extensive calcification. Patients with posterior circulation infarcts and severe middle cerebral artery stenosis were excluded. Cardioembolic stroke patients such as atrial fibrillation were also excluded.

### Standard ultrasound and CEUS examinations

Carotid standard ultrasound and CEUS examinations were performed within 72 h after admission. A high-resolution ultrasound instrument (Toshiba Medical Systems, Tokyo, Japan) equipped with a 12–15 MHz linear probe was used. The patients were lying supine, and the head turned slightly to the side contralateral to the carotid examined. Dual imaging mode was used to perform CEUS imaging and gray-scale imaging simultaneously in the same scanning plane. 2.5 mL ultrasound contrast agent (SonoVue, Bracco, Italy) by adding 5 mL saline was administered into a single median cubital vein in each patient. The mechanical index and gain were adjusted to visualize carotid plaque optimally (11). The continued images for at least 5 min were recorded in long-axis and short-axis views. The patient remained under observation for at least 30 min to ensure safety.

Carotid stenosis degree was measured based on existing guideline (12). On the standard ultrasound, plaque echogenicity was classified as uniformly hypoechoic (type I), predominantly hypoechoic (type II), predominantly hyperechoic (type III), and uniformly hyperechoic (type IV) (13). Plaques were further categorized as hypoechoic (Type I–II) or hyperechoic (Type III–IV) for clinical simplicity. Type V Plaques were calcified plaques. In this study, patchy calcification plaques with acoustic shadowing were excluded (14).

Intraplaque neovascularization was evaluated based on the extent of plaque enhancement. A visual semiquantitative grading scale was used to classify plaque enhancement degrees (15). Grade I: non-enhancement; grade II: arterial wall vasa vasorum enhancement; grade III: arterial wall vasa vasorum as well as plaque shoulder enhancement; grade IV: extensive and internal plaque enhancement. To explore the relationship between vessels within the plaque and cardiovascular risk factors, this study dichotomized CEUS findings into plaque neovascularization absent (grades I and II) and plaque neovascularization present (grades III and IV). Two independent observers reviewed a frame-by-frame image to ensure accuracy. Inconsistent gradings were discussed, and both observers determined the final result.

### Cardiovascular risk factors

Patients’ baseline characteristics and biochemical examination results were obtained within 72 h after hospital admission. The baseline characteristics included age, gender, weight, height, history of hypertension, diabetes, smoking history, and medication history. The biochemical examination contains low-density lipoprotein cholesterol (LDL-C), high-density lipoprotein cholesterol (HDL-C), total cholesterol (TC), triglyceride (TG), serum glucose concentration, white blood cell, C-reactive protein, and uric acid.

Hypertension: systolic blood pressure ≥ 140 mm Hg or diastolic blood pressure ≥ 90 mm Hg (16). According to 2013 ESC/ESH Guidelines for the management of arterial hypertension, patients with a history of antihypertensive treatment were included (17).

Dyslipidemia (18): LDL-C > 3.37 mmol/L, HDL-C < 1.04 mmol/L, TC > 5.18 mmol/L, or TG > 1.70 mmol/L. Abnormal concentrations of any or all of the above lipids in the plasma were defined as dyslipidemia.

Diabetes mellitus (19): fasting plasma glucose ≥7.0 mmol/L, 2-h plasma glucose ≥11.1 mmol/L. Patients whose serum glucose was within normal range but with a history of antidiabetic treatment were excluded.

Patients who had a 30 pack-year history of smoking were categorized as smokers (20).

### Statistical analysis

Data were analyzed using IBM SPSS Statistics for Windows, Version 22.0 (Armonk, NY: IBM Corp). Normally distributed continuous variables were presented as mean ± standard deviation (SD), and nonnormally distributed variables were presented as median (interquartile range). Categorical variables were presented as number (*n*) and percent (%). Regression analyses were utilized to assess risk factors associated with carotid plaque neovascularization. Patients were further divided into three groups according to the tertiles of serum glucose. Tertile 1: < 6.20 mmol/L (*n* = 52), Tertile 2: 6.20–13.35 mmol/L (*n* = 52), Tertile 3: > 13.35 mmol/L (*n* = 53). Multivariate analysis was also conducted to assess the relationship between serum glucose and plaque neovascularization. Tertile 1 was used as the referent group. Statistical significance was taken as *p* < 0.05.

## Results

### Baseline characteristics

**Figure 1:**
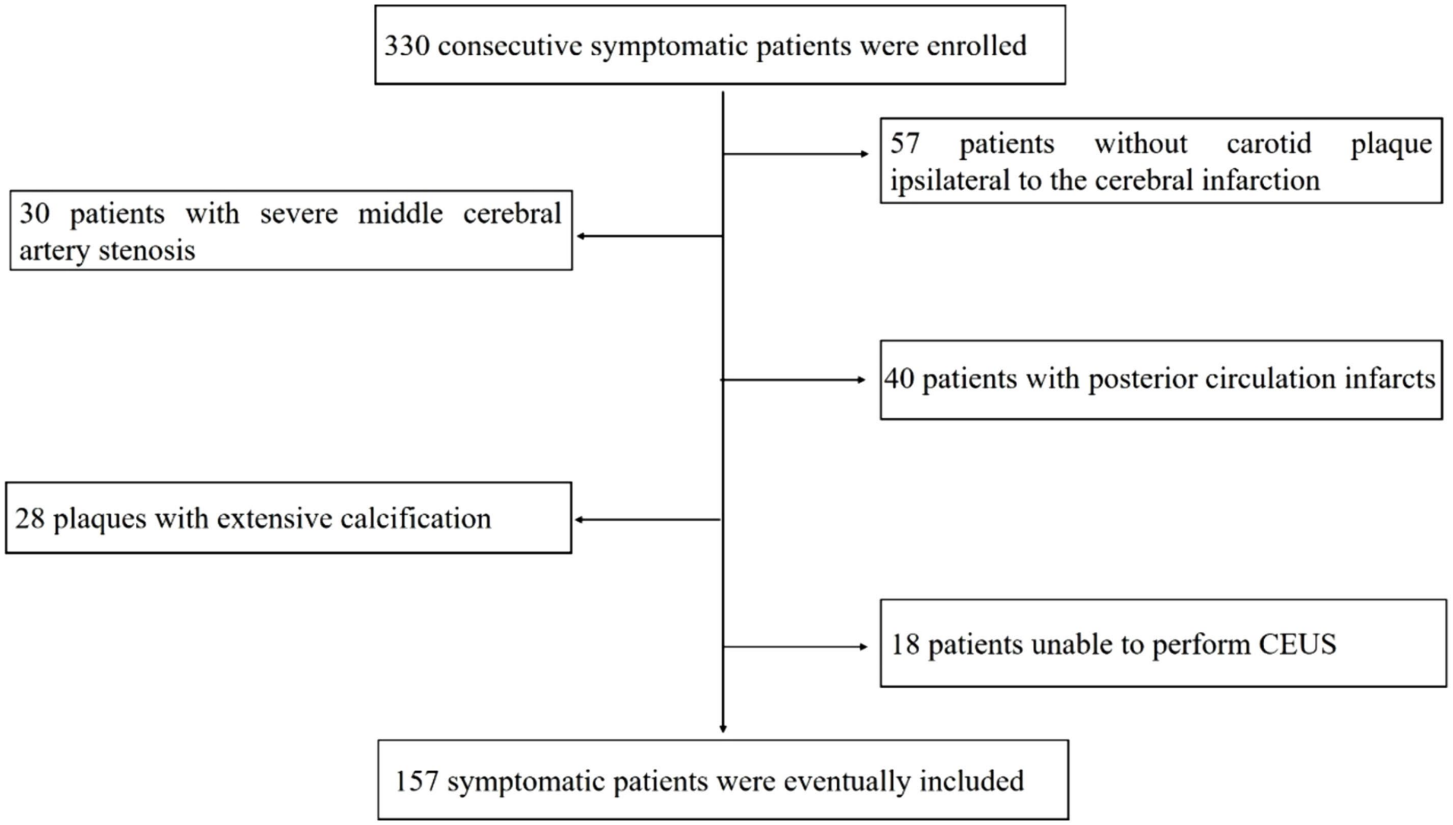
Flowchart of patients enrolled in the study.

330 consecutive symptomatic patients referred to the hospital were enrolled. After excluding 57 patients without carotid plaque ipsilateral to the cerebral infarction, 30 patients with severe middle cerebral artery stenosis, 40 patients with posterior circulation infarcts, 28 patients with extensive calcification plaques, 18 patients unable to perform CEUS imaging. 157 symptomatic patients were eventually included in the analysis (Figure 1).

The mean age was 67.0 (62.0, 70.0) years, and 133 (84.7%) were men. 72 (45.9%) patients had hypertension, 96 (61.1%) had dyslipidemia, and 92 (58.6%) had diabetes mellitus. 55 (35.0%) were smokers. 65 (41.4%) patients were treated with statins. Patients with intraplaque neovascularization were more prone to have diabetes mellitus (40.0% vs. 71.7%, *p* < 0.001), dyslipidemia (49.2% vs. 69.6%, *p* = 0.016) and hypertension (30.8% vs. 56.5%, *p* = 0.002). Male were more likely to have intraplaque neovascularization (76.9% vs. 90.2%, *p* = 0.040). Patients with intraplaque neovascularization had increased levels of C-reactive protein (*p* = 0.040). Use of statins were less common in patients with intraplaque neovascularization (55.4% vs. 31.5%, *p* = 0.005) (Table 1).

**Table 1:** Baseline variables in patients (*n* = 157).

Population characteristic	Total plaque (*n* = 157)	Neovascularization absent (*n* = 65)	Neovascularization present (*n* = 92)	*p*
Age (year)	67.0 (62.0, 70.0)	66.0 (60.0, 70.0)	67.0 (63.8, 70.2)	0.356
Gender (Male)	133 (84.7)	50 (76.9)	83 (90.2)	**0.040**
Hypertension	72 (45.9)	20 (30.8)	52 (56.5)	**0.002**
Systolic pressure	141.4 ± 19.1	137.2 ± 18.0	144.4 ± 19.3	**0.019**
Diastolic pressure	80.0 (70.0, 90.0)	77.0 (70.0, 88.0)	80.5 (70.8, 90.0)	0.616
Diabetes mellitus	92 (58.6)	26 (40.0)	66 (71.7)	**< 0.001**
Serum glucose	11.2 (5.6, 14.5)	5.8 (5.0, 12.0)	12.5 (7.3, 14.7)	**< 0.001**
Dyslipidemia	96 (61.1)	32 (49.2)	64 (69.6)	**0.016**
HDL-C	1.0 (0.9, 1.1)	1.0 (0.9, 1.1)	1.0 (0.9, 1.1)	**0.034**
LDL-C	3.2 (1.9, 5.7)	2.6 (1.9, 5.7)	3.5 (1.9, 5.7)	0.264
Tc	3.7 (3.1, 4.5)	3.7 (3.1, 4.3)	3.7 (3.1, 4.5)	0.818
Tg	1.2 (0.9, 1.6)	1.2 (0.9, 1.6)	1.3 (0.9, 1.7)	0.435
BMI (Kg/m^2^)	24.0 (22.4, 27.6)	23.9 (22.3, 26.7)	24.2 (22.5, 27.6)	0.638
Smoking	55 (35.0)	16 (24.6)	39 (42.4)	**0.033**
C-reactive protein	2.1 (1.6, 6.1)	1.8 (1.6, 2.7)	3.3 (1.4, 8.3)	**0.040**
White blood cell	6.6 (5.6, 7.8)	6.3 (5.5, 7.5)	6.6 (5.6, 7.8)	0.100
Uric acid	279.9 (234.8, 36.5)	297.7 (246.3, 368.2)	277.2 (220.8, 333.3)	0.113
Homocysteine	15.07 ± 8.3	15.5 ± 8.2	14.7 ± 8.5	0.152
Statin use	65 (41.4)	36 (55.4)	29 (31.5)	**0.005**
Anti-platelet use	87 (55.4)	40 (61.5)	47 (51.1)	0.257
Carotid stenosis				0.089
< 30%	44 (28.0)	23 (35.4)	21 (22.8)	
30–69%	83 (52.9)	34 (52.3)	49 (53.3)	
70–99%	30 (19.1)	8 (12.3)	22 (23.9)	
Plaque echogenicity				0.062
hypoechoic (Type1–2)	91 (58.0)	32 (49.2)	59 (64.1)	
hyperechoic (Type 3–4)	66 (42.0)	33 (50.8)	33 (35.9)	

Numbers are given as *n* (%), mean ± SD or median (interquartile range). BMI, body mass index; HDL-C, High-density lipoprotein cholesterol; LDL-C, Low-density lipoprotein cholesterol; TC, total cholesterol; TG, triglycerides. Significant results are displayed in bold.

### Standard ultrasound imaging and CEUS imaging

In the standard ultrasound examination, 40 (25.5%) plaques were type I, 51 (32.5%) plaques were type II, 31 (19.7%) plaques were type III, and 35 (22.3%) plaques were type IV. In conclusion, 91 (58.0%) plaques were hypoechoic (Type I–II) and 66 (42.0%) plaques were hyperechoic (Type III – IV). There was no difference in the plaque neovascularization among different plaque echogenicity (*p* = 0.062; Table 1).

**Figure 2:**
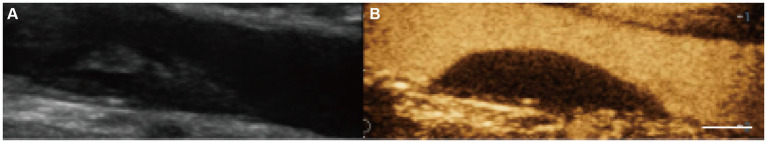
Plaque neovascularization absent. **(A)** Gray-scale ultrasound imaging demonstrated carotid hypoechoic plaque. **(B)** During CEUS imaging, no bubbles within the plaque or adventitia enhancement (scale bar: 1 cm).

**Figure 3:**
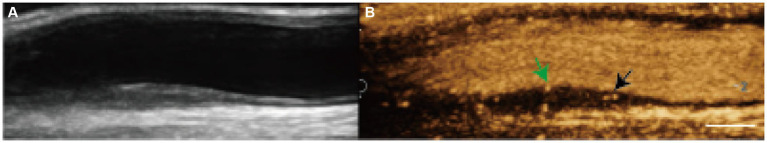
Plaque neovascularization present. **(A)** Gray-scale ultrasound imaging demonstrated carotid hyperechoic plaque. **(B)** During CEUS imaging, moving bright spots of microbubbles were seen within the carotid plaque shoulder (black arrow) and core (green arrow) (scale bar: 1 cm).

In the CEUS imaging, 65 (41.4%) plaques did not appear intraplaque neovascularization, while 92 (58.6%) plaques presented intraplaque neovascularization (Figures 2, 3). A higher prevalence of intraplaque neovascularization was demonstrated in hypoechoic plaques, but remained no statistical significance (*p* = 0.062) (Table 1).

44 (28.0%) plaques were mild carotid stenosis, 83 (52.9%) plaques were moderate stenosis, and 30 (19.1%) lesions were severe stenosis. However, no correlation existed among carotid stenosis and plaque neovascularization (*p* = 0.089) (Table 1).

### Correlation between plaque neovascularization and risk factors

In Univariate analysis, diabetes mellitus (OR 3.81, 95% CI 1.94–7.46, *p* < 0.001), and dyslipidemia (OR 2.36, 95% CI 1.22–4.55, *p* = 0.011) were risk factors for plaque neovascularization. The risk of having plaque neovascularization was increased in smoking patients (OR 2.25, 95% CI 1.12–4.54, *p* = 0.023). Treatment with statins was significantly lower in patients with plaque neovascularization (OR 0.37, 95% CI 0.19–0.72, *p* = 0.003) (Table 2).

**Table 2:** Associations between risk factors and plaque neovascularization (*n* = 157).

Variable	Univariate		Multivariate Model 1		Multivariate Model 2	
	OR (95% CI)	*p*	OR (95% CI)	*p*	OR (95% CI)	*p*
Hypertension	2.92 (1.50–5.71)	**0.002**	2.79 (1.40–5.54)	**0.003**	0.93 (0.32–2.71)	0.894
Diabetes	3.81 (1.94–7.46)	**<0.001**	3.88 (1.95–7.70)	**<0.001**	3.27 (1.10–9.78)	**0.034**
Dyslipidemia	2.36 (1.22–4.55)	**0.011**	2.31 (1.19–4.52)	**0.014**	1.14 (0.51–2.55)	0.752
Smoking	2.25 (1.12–4.54)	**0.023**	2.07 (1.01–4.24)	**0.047**	1.28 (0.58–2.84)	0.543
Statin	0.37 (0.19–0.72)	**0.003**	0.38 (0.19–0.74)	**0.004**	0.49 (0.23–1.04)	0.063
C-reactive protein	1.11 (1.03–1.20)	**0.008**	1.10 (1.02–1.19)	**0.015**	1.06 (0.97–1.15)	0.213

Model 1: each variable was adjusted for age and gender. Model 2: final multivariate model with age, gender and significant model 1 variables. CI, confidence interval; OR, odds ratio. Significant results are displayed in bold.

After adjusted for age and gender, regression analysis Model 1 revealed that plaque neovascularization was positively associated with diabetes mellitus (OR 3.88, 95% CI 1.95–7.70, *p* < 0.001), dyslipidemia (OR 2.31, 95% CI 1.19–4.52, *p* = 0.014), and smoking (OR 2.07, 95% CI 1.01–4.24, *p* = 0.047). The positive association between plaque neovascularization and C-reactive protein (OR 1.10, 95% CI 1.02–1.19, *p* = 0.015) remained statistically significant. However, plaque neovascularization was negatively associated with statin treatment (OR 0.38, 95% CI 0.19–0.74, *p* = 0.004) (Table 2).

Regression analysis Model 2 was also performed after adjusting for various significant confounders. The multivariate analysis showed that diabetes mellitus (OR 3.27, 95% CI 1.10–9.78, *p* = 0.034) was an independent risk factor related to plaque neovascularization (Table 2).

### Correlation between serum glucose and plaque neovascularization

Univariate regression analysis was performed in patients with different tertiles of serum glucose. Compared to the patients in the first tertile of serum glucose (< 6.20 mmol/L), patients in the second quartile (6.20–13.35 mmol/L) and patients in the third quartile (> 13.35 mmol/L) were associated with an increased risk of having plaque neovascularization (Table 3).

**Table 3:** Univariate and multivariate logistic regression of association between serum glucose and plaque neovascularization.

Variable	Univariate		Multivariate Model 1		Multivariate Model 2	
	OR (95% CI)	*p*	OR (95% CI)	*p*	OR (95% CI)	*p*
Quartile 1	Ref		Ref		Ref	
Quartile 2	4.24 (1.87–9.62)	**0.001**	4.1 (1.78–9.45)	**0.001**	3.86 (1.41–10.53)	**0.008**
Quartile 3	6.33 (2.7–14.86)	**<0.001**	7.05 (2.91–17.08)	**<0.001**	5.55 (1.85–16.66)	**0.002**

Model 1: adjusted for age and gender. Model 2: adjusted for age, gender, hypertension, dyslipidemia, smoking, statin and C-reactive protein. Tertile 1: < 6.20 mmol/L (*n* = 52), Tertile 2: 6.20–13.35 mmol/L (*n* = 52), Tertile 3: > 13.35 mmol/L (*n* = 53). CI, confidence interval; OR, odds ratio. Significant results are displayed in bold.

In the multivariate regression analysis, compared to the patients in the first tertile of serum glucose (< 6.20 mmol/L), the patients in the third quartile (> 13.35 mmol/L) had a significantly higher risk of having plaque neovascularization (OR 5.55, 95% CI 1.85–16.66, *p* = 0.002) (Table 3).

## Discussion

The current study examined the association between cardiovascular risk factors and intraplaque neovascularization in symptomatic patients. The findings indicated that diabetes, dyslipidemia, smoking, hypertension, and elevated C-reactive protein levels are significant risk factors for plaque neovascularization. Conversely, the use of statins may serve as a protective factor against this condition. After adjusting for multiple confounding variables, diabetes mellitus emerged as an independent risk factor significantly correlated with plaque neovascularization, suggesting potential therapeutic implications.

Contrast-enhanced ultrasound (CEUS), a non-invasive imaging modality that has garnered significant utilization in recent years, is employed to evaluate neovascularization within atherosclerotic plaques. Empirical evidence suggests a robust correlation between carotid plaque enhancement as visualized by CEUS and the clinical manifestations in patients with atherosclerosis (21). Notably, elevated plaque enhancement is indicative of increased neovascularization and heightened plaque instability (22). Furthermore, contrast-enhanced ultrasound (CEUS) not only enhances the early detection of vulnerable carotid plaques but also assess the prognosis of the patients by identifying neovascularization within the plaques. This capability facilitates the examination of prior cardiovascular and cerebrovascular events, the identification of cerebrovascular risk, and the prediction of future cardiovascular and cerebrovascular events. Recent studies have demonstrated that patients with grade 2 carotid intraplaque neovascularization, as assessed by CEUS, are more likely to experience future cardiovascular events compared to those with lower grades of neovascularization (23).

Diabetes mellitus has emerged as a significant global health concern in recent years. It is well-established that diabetes is a critical risk factor for atherosclerotic disease, with an increased prevalence of carotid atherosclerotic plaques observed in diabetic patients. Recent studies have further elucidated the connection between diabetes mellitus and the vulnerability characteristics of atherosclerotic plaques. For instance, Sugiyama et al. (24) conducted a study involving 322 patients with acute coronary syndromes, utilizing optical coherence tomography imaging to examine coronary artery plaques. Their findings indicated that plaques in diabetic patients exhibited more vulnerable features. Sun et al. (25) conducted an evaluation of 140 carotid plaques and concluded that lipid-rich plaques were significantly more prevalent in diabetic patients experiencing acute cerebrovascular syndromes. Additionally, Moreno et al. (26) observed a significant increase in lipid core content and macrophage infiltration in the coronary plaques of diabetic patients. Purushothaman et al. (27) conducted a study on 230 plaques from patients with diabetes mellitus and found a significant increase in inflammatory cells, specifically macrophages and T-lymphocytes, in these patients. Recent research has also indicated that intraplaque neovascularization is common in patients with intermediate carotid stenosis and is more prevalent among those with diabetes (28). Consistent with prior research, our study identified diabetes mellitus as an independent risk factor associated with plaque neovascularization. We excluded patients with posterior circulation infarcts, severe middle cerebral artery stenosis, and atrial fibrillation to ensure that the strokes analyzed were confined to the internal carotid artery territory. After adjusting for potential confounders, the association between diabetes and intraplaque neovascularization remained statistically significant. Additionally, we conducted multivariate logistic regression analyses on patients stratified into different tertiles of serum glucose levels. In comparison to patients in the first tertile of serum glucose (< 6.20 mmol/L), those in the third quartile (> 13.35 mmol/L) exhibited the most pronounced intraplaque neovascularization. Consequently, patients with serum glucose levels exceeding 13.35 mmol/L may be at an elevated risk for intraplaque neovascularization.

Intraplaque neovascularization is a hallmark of plaque vulnerability. The complex interaction among various risk factors in the development of neovascularization constitutes a significant contributor to plaque vulnerability. Endothelial proliferation stimulated by VEGF is a key factor in this process (29). Endothelial dysfunction induced by chronic hyperglycemia and insulin resistance may promote the secretion of vascular endothelial growth factors (VEGF) (30). Under hyperglycemic conditions, the overexpression of VEGF can play a crucial role in microangiopathy and enhance the angiogenic response. Moreover, basic fibroblast growth factor (bFGF), VE-cadherin, E-selectin, intercellular adhesion molecule-1 (ICAM-1), and vascular cell adhesion molecule-1 (VCAM-1) have been identified as key factors in promoting endothelial cell proliferation, thereby contributing to the development of permeable neovessels (31). Inflammatory infiltration may represent an additional mechanism by which hyperglycemia-induced oxidative stress leads to increased neovascularization. Recent study has observed that neovascularization frequently occurred alongside the inflammatory response, characterized by a marked infiltration of macrophages and T-lymphocytes (32). T cells and macrophages express chemokine receptors, including CXCL-9, CXCL-10, and CXCL-5, which facilitate the recruitment of circulating cells into the vascular layer (33). Immune responses driven by T helper cells, potentially mediated by interferon-γ, may inhibit smooth muscle cell proliferation and contribute to medial disruption and the paucity of smooth muscle cells in penetrating neovessels (34). The development of microvasculature within plaques may be significantly influenced by the combined effects of hyperglycemia-induced overexpression of vascular endothelial growth factor and inflammatory infiltration.

The current study further demonstrated that C-reactive protein levels were significantly elevated in patients with carotid plaque neovascularization. After adjusting for age and gender, an increased C-reactive protein level remained statistically associated with extensive plaque enhancement. C-reactive protein (CRP) is widely recognized as a crucial biomarker of inflammation implicated in the progression of atherosclerosis (35). Alvarez et al. (36) reported that elevated serum CRP levels were associated with increased T lymphocyte and macrophage content in carotid plaques. Additionally, serum CRP levels were positively correlated with the vulnerability of carotid plaques. Several studies have implicated CRP in the direct stimulation of angiogenesis, thereby reinforcing the connection between the inflammatory response and angiogenesis (37). Among these factors, pro-inflammatory cytokines were implicated in angiogenic responses. The early stages of neovascularization were characterized by an active inflammatory process surrounding the lumen, facilitated by leukocyte adhesion and migration (38). Our data suggest that increased neovascularization within the plaque is associated with a more pronounced inflammatory response, potentially contributing to the transition of a stable carotid plaque to a vulnerable state.

Lesions exhibiting intraplaque neovascularization were significantly more prevalent in patients with smoking habits, indicating that smoking may expedite the progression of intraplaque neovascularization. Currently, the precise mechanism by which smoking elevates the risk of plaque neovascularization remains undetermined. Smoking is recognized as one of the most critical modifiable risk factors for atherosclerosis. It has been well-documented that even light smoking can induce inflammatory infiltration (39). The development of inflammatory infiltration may act as an initiator in the onset of angiogenesis. Additionally, prolonged cigarette smoking has been linked to a diminished capacity of the blood to deliver oxygen (40). Hypoxia could potentially accelerate angiogenesis. Although smoking was not identified as an independent risk factor for extensive enhancement in multivariate regression analysis, we observed more extensive enhancement of plaques in patients with smoking habits. Consequently, the impact of cigarette smoking on angiogenesis warrants further investigation.

Previous research has documented that statin treatment reduces intraplaque angiogenesis in carotid endarterectomy specimens (41). Our findings are consistent with these prior studies, demonstrating a significant decrease in statin treatment among patients with CEUS grade 2 plaques. While statins are well-established for their lipid-lowering effects and their role in suppressing atherosclerosis, their additional benefits in reducing intraplaque angiogenesis are not yet fully understood. Furthermore, statins exhibit an inhibitory effect on lipoprotein oxidation, attributable to their antioxidant properties (42). Furthermore, the anti-inflammatory properties of statins may significantly contribute to plaque stability by diminishing leukocyte-endothelial cell adhesion and reducing the levels of inflammatory cytokines (43). The pleiotropic anti-inflammatory and anti-oxidative effects of statins are likely mechanisms underlying the inhibition of angiogenesis, which may impact plaque neovascularization by preventing cholesterol accumulation and the infiltration of monocyte-derived macrophages.

In addition to traditional risk factors such as hypertension, hyperlipidemia, hyperglycemia, smoking, and alcohol consumption, emerging risk factors have also been identified in the incidence of ischemic stroke. Notably, research on homocysteinemia has garnered significant attention. Studies have demonstrated that hyperhomocysteinemia is an independent risk factor for cerebral infarction and may serve as an early diagnostic marker for this condition (44). Previous research has indicated that approximately one-third of stroke patients exhibit hyperhomocysteinemia (45). This study specifically included symptomatic patients with anterior circulation infarcts. Within this cohort, no significant correlation was observed between the presence and absence of plaque neovascularization. This lack of correlation may be attributable to variations in patient recruitment criteria. The regulation of the specific mechanism underlying hyperhomocysteinemia is a multifaceted process potentially involving the activity of various enzymes and is influenced by factors such as diet, cellular stress, genomic background, and the epigenetic environment. The pathogenic mechanism remains inadequately understood and warrants further elucidation. Given that this study employs a cross-sectional design, subsequent research should focus on conducting a longitudinal cohort study to investigate the role of homocysteine in stroke recurrence.

### Limitations

Firstly, the number of included patients was relatively small. In addition, this study evaluated symptomatic patients at least with one carotid plaque ipsilateral to the anterior cerebral circulation infarction. Consequently, this study may not reflect all high-risk atherosclerotic patients. Lastly, this study investigated the influence of a single risk factor on plaque neovascularization. Although diabetes mellitus was independently related to plaque neovascularization, it is possible that multiple risk factors interact to enhance their effects. In order to fully understand the impact of combining two or more risk factors on plaque vulnerability progression, further research is needed.

## Conclusion

The current study demonstrated that diabetes mellitus serves as an independent high-risk factor for carotid intraplaque neovascularization, potentially influencing the vulnerability of carotid plaques. From a clinical perspective, effective glycemic control may enhance risk assessment and facilitate personalized medical interventions.

## Data Availability

The raw data supporting the conclusions of this article will be made available by the authors, without undue reservation.
